# Exploration of sleep function connection and classification strategies based on sub-period sleep stages

**DOI:** 10.3389/fnins.2022.1088116

**Published:** 2023-01-25

**Authors:** Fangzhou Xu, Jinzhao Zhao, Ming Liu, Xin Yu, Chongfeng Wang, Yitai Lou, Weiyou Shi, Yanbing Liu, Licai Gao, Qingbo Yang, Baokun Zhang, Shanshan Lu, Jiyou Tang, Jiancai Leng

**Affiliations:** ^1^International School for Optoelectronic Engineering, Qilu University of Technology (Shandong Academy of Sciences), Jinan, China; ^2^School of Mathematics and Statistics, Qilu University of Technology (Shandong Academy of Sciences), Jinan, China; ^3^Department of Neurology, Shandong Institute of Neuroimmunology, Shandong Key Laboratory of Rheumatic Disease and Translational Medicine, The First Affliated Hospital of Shandong First Medical University, Shandong Provincial Qianfoshan Hospital, Jinan, China; ^4^Department of Neurology, Cheeloo College of Medicine, Shandong Qianfoshan Hospital, Shandong University, Jinan, Shandong, China

**Keywords:** electroencephalography (EEG), sleep stage, classification, brain functional connectivity, phase-locked value (PLV)

## Abstract

**Background:**

As a medium for developing brain-computer interface systems, EEG signals are complex and difficult to identify due to their complexity, weakness, and differences between subjects. At present, most of the current research on sleep EEG signals are single-channel and dual-channel, ignoring the research on the relationship between different brain regions. Brain functional connectivity is considered to be closely related to brain activity and can be used to study the interaction relationship between brain areas.

**Methods:**

Phase-locked value (PLV) is used to construct a functional connection network. The connection network is used to analyze the connection mechanism and brain interaction in different sleep stages. Firstly, the entire EEG signal is divided into multiple sub-periods. Secondly, Phase-locked value is used for feature extraction on the sub-periods. Thirdly, the PLV of multiple sub-periods is used for feature fusion. Fourthly, the classification performance optimization strategy is used to discuss the impact of different frequency bands on sleep stage classification performance and to find the optimal frequency band. Finally, the brain function network is constructed by using the average value of the fusion features to analyze the interaction of brain regions in different frequency bands during sleep stages.

**Results:**

The experimental results have shown that when the number of sub-periods is 30, the α (8–13 Hz) frequency band has the best classification effect, The classification result after 10-fold cross-validation reaches 92.59%.

**Conclusion:**

The proposed algorithm has good sleep staging performance, which can effectively promote the development and application of an EEG sleep staging system.

## 1. Introduction

With the development of society, more and more people have the problem with sleep disorders, and how to diagnose and intervene in early sleep disorders is particularly important (Miyata et al., 2007; Younes, 2017). Although electroencephalography (EEG), electrocardiogram (ECG), electromyogram (EMG), and other physiological signals can be used for sleep staging (Yan et al., 2019), EEG signals contain more information and can better reflect the overall information. Sleep staging is the basis of sleep quality assessment. The traditional EEG sleep stage division still requires sleep experts to manually divide according to special brain waves and duration (Ronzhina et al., 2012). The procedure is time-consuming, laborious, and subject to subjective errors (Chapotot and Becq, 2010). The automatic sleep staging method plays an important role in the early diagnosis and intervention of sleep disorders.

In 1968, R&K (Wolpert, 1969) divided sleep into awake, rapid eye movement (REM), and non-rapid eye movement (NREM) stages, of which NREM is further subdivided into four stages: S1, S2, S3, and S4. Because the S3 and S4 stages are similar, the American Academy of Sleep Medicine (AASM) (Berry et al., 2012a) modified the R&K rule, which used N1, N2, and N3 to represent the different stages of NREM, and merged S3 and S4 into the N3 stage. Most studies interpret sleep stages sequentially according to the 30 s recording frame, and if the 30 s is divided into multiple segments, some unique features may be found. In the study of Diykh and Li (2016), the authors divided the EEG signal period of the 30 s into 75 sub-periods and then extracted 12 statistical features from each sub-period. This study achieved 92% classification of 6 sleep stages. Seo et al. (2020) used a convolutional neural network (CNN) to extract features from signal sub-bands and used bi-directional long short-term memory (BiLSTM) to learn the temporal context of representative features. The features learned from continuous signal sub-bands in this study can represent the temporal characteristics of EEG signals, but important sleep-related events may only appear in some special sub-bands (Weber et al., 2021), so it is also necessary to consider the temporal characteristics of EEG signals. Characteristics of learning different brain activities in signaling sub-bands. An et al. (2021) mapped multiple signal wavelets to the amplitude axis and the time axis, respectively, and extracted statistical classification features from the mapped feature information, The accuracy of the classification of sleep stages 5 and 6 sleep stages reached 89.18 and 88.42%. In summary, the key to EEG sleep staging is how to obtain effective classification features and find optimal features from EEG signals in multiple sub-periods.

To obtain effective classification features, researchers have proposed many traditional feature extraction methods, which are divided into the following four types: (1) time domain features. (2) frequency domain features. (3) time-frequency domain features. (4) nonlinear features. (Gunnarsdottir et al., 2018) extracted time-domain and frequency-domain features from PSG signals, using data from healthy people, and using a decision table classifier to classify the extracted attributes, with an overall classification accuracy of 80.70%. da Silveira et al. (2016) used discrete wavelet transform techniques to analyze the changes in sleep behavior in different frequency ranges, extracted skewness, kurtosis, and variance features from the corresponding input channels, and evaluated the ability of random forest classifiers to distinguish different sleep stages. Tests were carried out and the results showed an overall accuracy of 90%. Zhu et al. (2014) proposed a sleep stage classification method based on the time and frequency domain features of single-channel EEG signals. EEG signals were mapped onto visibility maps and level maps to detect gait-related movements, and the nine features extracted from the input signal were forwarded to the support vector machines (SVM) classifier that considers multiple sleep stages. The method achieved 87.50% accuracy for the two-state sleep stage classification problem. Tabar et al. (2021) used a bootstrapping method guided by mutual information to partition sleep stages into a low-dimensional feature space and used fewer features to classify sleep stages. In recent years, deep learning methods have been widely used in sleep stage classification. Seo et al. (2020) proposed a deep learning model intra- and inter-epoch temporal context network (IITNet), for learning intra and inter-epoch temporal context from raw single-channel EEG for automatic sleep scoring, this model has been tested on the Sleep-EDF, Montreal Archive of Sleep Studies (MASS), and Sleep Heart Health Study (SHHS) datasets and obtained the accuracies of 83.9, 87.2, and 86.7%. Mousavi et al. (2019) proposed a network architecture including 9 convolutional layers and 2 fully connected layers to extract features from raw EEG signals, this automatic identification method used single-channel EEG signals to classify 2–6 sleep-like stages. Khalili and Asl (2021) used a CNN to extract features and then employed a temporal convolutional neural network to extract temporal features from the feature vector extracted by CNN, respectively, in Sleep-EDF-2013 and Sleep-EDF-2018 two datasets got 85.39 and 82.46% classification accuracy.

Most of the existing feature extraction methods extract features from a single channel (Terzano et al., 2001; Tagliazucchi et al., 2013; Tagliazucchi and Laufs, 2014; Lv et al., 2015; Desjardins et al., 2017; Stevner et al., 2019; Fu et al., 2021), the calculation is also performed separately on a single channel. The amount of information obtained through a single channel does not fully characterize the changes in brain activities during sleep, making it difficult to explore sleep stage information from a global level. Current research mainly uses functional magnetic resonance imaging (fMRI) to analyze brain function in different brain regions. The fMRI has confirmed that each sleep stage is associated with specific functional connectivity patterns (Goldberger et al., 2000; Berry et al., 2012b; Brignol et al., 2012). Brain functional network is a relatively new measure to characterize the exchange of information between brain regions by calculating the temporal correlation or coherence between them (Baptista et al., 2010; Siettos and Starke, 2016; Rattenborg et al., 2020). EEG-based brain functional connectivity is increasingly being used in sleep studies to differentiate sleep disorders from healthy individuals (Liu et al., 2010; Gao et al., 2015; Guo et al., 2019). Functional connectivity is employed to explore synchronization mechanisms between different brain regions and sleep stage classification accuracy. At present, the common methods to analyze brain functional connections are phase-locked value (PLV) (Diykh et al., 2018), directional transfer function (DTF) (Brázdil et al., 2009), coherence (Bortel and Sovka, 2006), granger causality analysis (GCA) (Chen et al., 2019), and mutual information (MI) (Caballero-Gaudes et al., 2013). PLV is a classical method to construct a functional network. PLV is only sensitive to phase but not amplitude and is often used to measure the phase synchronization between two signals. Compared with other synchronization measures, PLV is easy to operate and can keep the same information level as more complex indicators. In this manuscript, PLV is used to construct brain networks in different sleep stages.

**FIGURE 1:**
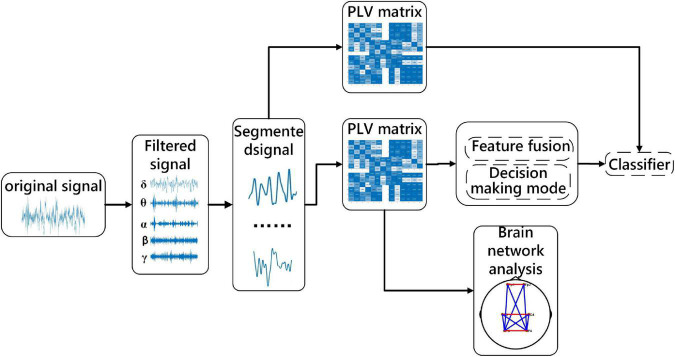
The flow diagram of the proposed framework.

The functional network is used to analyze the brain interaction and connection mechanism in different sleep stages. In this manuscript, the methods of multi-sub-periods and different frequency bands are proposed to decode EEG signals in different sleep stages. The main contributions are as follows,

(1) The PLV method is used to find the optimal frequency band and sub-period numbers. The experimental results have shown that when the number of sub-periods is 30, the α (8–13 Hz) frequency band has the best classification performance.

(2) The multiple sub-periods are used for feature fusion. The classification performance optimization strategy is used to obtain an accuracy of 92.59%.

(3) In the optimal frequency band, a functional connection network is constructed to explore the brain interaction and connection mechanism in different sleep stages. The specific process is shown in Figure 1.

The rest of this study is organized as follows. Sections “2. Materials” and “3. Methods” describe the materials and methods. Section “4. Experimental results and analysis” shows all results. Sections “5. Discussion” and “6. Conclusion” provide a discussion and a summary of future work, respectively.

## 2. Materials

This manuscript uses the Cyclic Alternating Pattern (CAP) sleep database (Diykh et al., 2016; Yüce and Yaslan, 2016), available on the PhysioNet website. There are 108 different subjects of sleep diseases and health in CAP database, including 92 sleep disorders subjects and 16 healthy subjects. The dataset includes at least 3 EEG channels, EOG, EMG, bilateral anterior tibial EMG, respiratory signal, and ECG. The sampling frequency is 512 Hz To better analyze the relationship between sleep state and brain regions, the calculation of functional brain network connectivity requires as many channels as possible. According to the international 10–20 system, FP2-F4, C4-P4, P4-O2, FP1- F3, C3-P3, P3-O1, and other 6 channels are analyzed. Because some subjects have no polysomnography data and reduce the influence of age on brain connectivity. Four healthy subjects and six nocturnal patients with frontal lobe epilepsy are used for analysis. These subjects are No. 3, No. 5, No. 10, and No. 11 in the healthy group and No. 3, No. 6, No. 11, No. 15, No. 16, and No. 21 in the nfle group. According to the latest sleep rules, S3 and S4 sleep stages are combined into the N3 sleep stage. The basic information on these subjects and epochs will be shown in Table 1.

**TABLE 1:** Basic information about each subject and period numbers of each stage.

	Condition	Age	N1	N2	N3	R	W	All stage	All time
Sub1	Healthy	35	49	347	279	188	136	999	499.5 min
Sub2	Healthy	35	49	413	303	232	10	1,007	503.5 min
Sub3	Healthy	23	2	261	308	215	67	853	426.5 min
Sub4	Healthy	28	6	266	344	380	56	1,052	526 min
Sub5	Nfle	29	72	419	209	261	136	1,097	548.5 min
Sub6	Nfle	32	37	323	236	190	24	810	405 min
Sub7	Nfle	31	28	320	366	279	27	1,020	510 min
Sub8	Nfle	29	19	417	274	227	97	1,034	517 min
Sub9	Nfle	30	9	398	131	152	109	799	399.5 min
Sub10	Nfle	27	31	209	257	254	78	801	400.5 min

## 3. Methods

### 3.1. Data preprocessing and channel selection

During human sleep, the stages of sleep change gradually, and there are no clear boundaries between different stages of sleep. This manuscript uses the data of the CAP database to segment the EEG into 30-s segments. For sleep stages, the adopted 30-s period comes from the R&K and AASM rules (Diykh et al., 2018), and related work has also revealed that 30-s period lengths are feasible for characterizing intrinsic brain activities (Phan et al., 2018; Zhou et al., 2020). The 30-s data is divided into 5-Sub (6 s), 10-Sub (3 s), 15-Sub (2 s), 20-Sub (1.5 s) and 30-Sub (1 s) methods. In addition, the data are filtered in five frequency bands, namely δ (0.5–4 Hz), θ (4–8 Hz), α (8–13 Hz), β (13–30 Hz), and γ (30–40 Hz).

In this manuscript, the adaptive channel selection algorithm in gradient boosting (GB) classifier has been proposed to achieve the optimal channel selection. The data of 12 channels in the CAP database have been connected in a one-to-one way. Different thresholds in the matrix have been set to find channels with good connectivity. Five different numbers of EEG channels, which include 12 channels, 10 channels, 8 channels, 6 channels, and 4 channels, have been selected for comparison. The 10-fold cross validation has been employed to verify the validation of the proposed algorithm.

### 3.2. Multi-subsegment strategy

**FIGURE 2:**
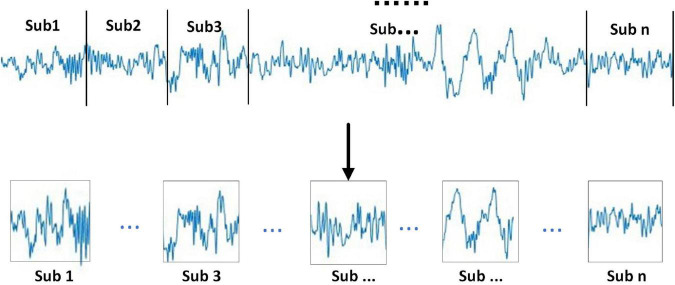
Sub-period division method direct PLV feature fusion decision choice.

It can be seen from Figure 2 that the specific steps of the multi-sub-segment strategy are divided the 30-s signal into multiple consecutive signal sub-periods, the divided multi-sub-periods do not overlap. In addition, the divided sub-periods are divided into sub-periods of the same length according to the sample size of the 30-s signal, as shown in Figure 2.

Let *L* be a 30-s sleep EEG data sample with a length of (30*512). When the number of sub-periods is set to be long *L*s, the length of the divided sub-periods satisfies the following constraints:


(1) L=L⁢s*N⁢s

*Ns* is the number of sub-periods divided by the sleep period. *L* is the sub-period length. The sampling frequency in the CAP sleep database is 512 Hz, and the length of the data sample is 30 s, so the specific calculation of the length of the sub-period can be as follows,


(2) L⁢s=L/N⁢s=(30*512)/N⁢s=15360/N⁢s

According to the above formula, the number of sub-periods *Ns* plays an important role in the experiment. By setting an appropriate number of sub-periods, effective classification features can be obtained from the divided sub-periods, and at the same time, redundancy can be properly handled. In addition, the division of multiple sub-periods is also related to the computational complexity of feature extraction, the features extracted from consecutive sub-periods have time-series features for analyzing sleep signals. 30-s data has been divided into sub-period data. In general, 30-s data can be divided into 30 sub-periods. The sleep stages of sub-periods have been recognized by the proposed algorithm. 30 classification results can be obtained from the 30 sub-periods. The final classification result of a 30-s data can be obtained from the highest result from the sub-periods. The classification is at this stage, and different numbers of sub-segments are used for comparative experiments to improve the overall classification results.

### 3.3. Phase lock value

For network analysis of sleep signals, the construction of the brain network is the basis of research and is crucial for network analysis. In this manuscript, the processed data are used to construct the brain network of sleep signals. The nodes involved in the brain network refer to the electrodes used in the data acquisition procedure, the connection between the networks refers to the functional connection between any two nodes (this study mainly considers the functional connection network). According to whether the flow of information between nodes is concerned, the constructed brain network can be divided into a directed network and an undirected network. There are many ways to build a network. For the construction of directed networks, the commonly used methods mainly include directional transfer function, granger causality, partial directional coherence, and so on. There are also many methods for constructing undirected networks, such as correlation, coherence, phase locking, and phase lag.

This manuscript adopts PLV to assess brain functional connectivity (Lachaux et al., 1999). PLV is widely used to measure the phase synchronization between each pair of electrodes. The reason is that PLV is only sensitive to the phase. Compared with other methods, the PLV method is simple to operate. PLV is the comparison between channel *i* and channel *j*. A high PLV value indicates a strong coupling relationship between the pair of electrodes (Quiroga et al., 2010). Therefore, PLV is used to construct the corresponding brain network in this manuscript. The calculation formula of PLV is defined as follows,


(3) $$P\,L\,V=\left|\frac{1}{N}\,\sum_{\text{j}=0}^{N-1}{\text{e}}^{i\,\Delta \,\Phi \,\left(t\right)}\right|\,\Delta \,\phi \,\left(t\right)=\phi_{x}\,\left(j\,\Delta \,t\right)-\phi_{y}\,\left(j\,\Delta \,t\right)$$

Among them, *N* represents the number of samples of the sleep signal, and *t* represents the sampling period, the two-time series are (*x*)*t* and (*y*)*t*, the two instantaneous phases are ϕ_*x*_ (t) and ϕ_*y*_ (t). Six EEG channels are used in the experiment, and a 6*6 PLV symmetric matrix has been obtained from each period, and each value in the matrix represented the coupling relationship of a pair of channels. In addition, brain network analysis between sleep stages is compared with the PLV matrix. The PLV matrices for each sleep stage are averaged and brain networks are constructed based on thresholds. The threshold is chosen from the maximum value that does not appear outlier in the network.

### 3.4. Fusion strategy

**FIGURE 3:**
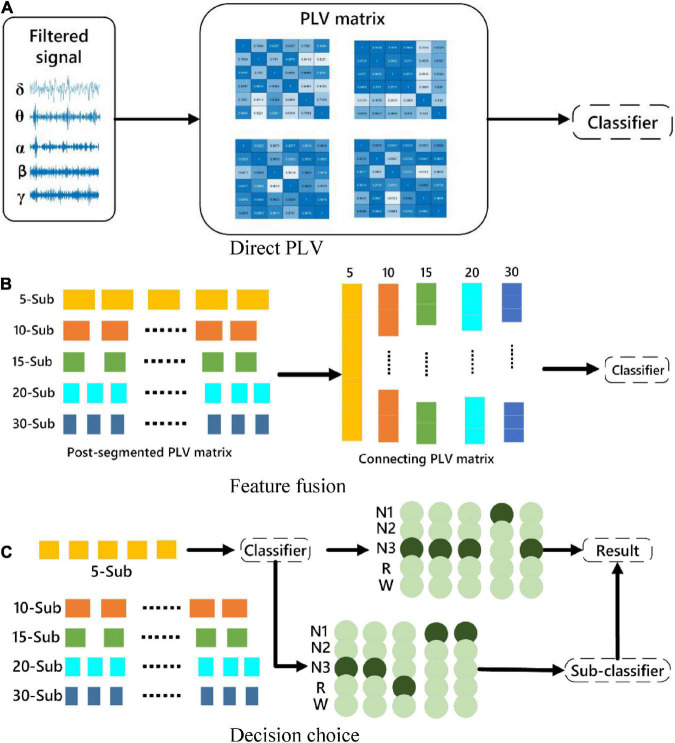
Schematic diagram of the classification strategy. **(A)** The original signal is directly subjected to PLV for classification. **(B)** Feature-level fusion for classification. **(C)** Multi-sub-segment classification for decision selection.

For the assessment of brain functional connectivity in different frequency bands and different numbers of sub-periods, three strategies of feature processing are used to classify sleep stages. The features mentioned here are the brain functional connectivity features extracted from the processed data. The three methods are, (1) Directly extract functional connectivity features from the 30-s EEG data. (2) Stack the functional connectivity features extracted from multiple sub-periods, and then input the features as a whole into the classifier. (3) Directly analyze the functional connectivity features of sub-periods Perform classification, and then take the mode of the classification result as the result of the entire 30-s segment. The specific description is shown in Figure 3. Divide the 30-s data into 5, 10, 15, 20 and 30 segments according to Section “3.1. Data preprocessing and channel selection.” The proposed three methods are used to experiment with the segmented data.

Because method C may result in the same number of classifications for certain two categories, a sub-classifier is designed to re-extract the data with the same classification results, transfer it to the sub-classifier, perform binary classification, and use the binary classification result as the final classification result.

### 3.5. Classifier

This manuscript adopts a support vector machine (SVM) with a Gaussian kernel function, which is implemented in the LibSVM library (Cortes and Vapnik, 1995; Chang and Lin, 2000). The way to achieve multi-class classification is to use a one-to-one strategy. Classification performance is evaluated from sleep stage accuracy for three strategies of classification across frequency bands. 80% of the samples are used for model training, the remaining 20% are used as test data.

## 4. Experimental results and analysis

### 4.1. Channel selection and band comparison

**FIGURE 4:**
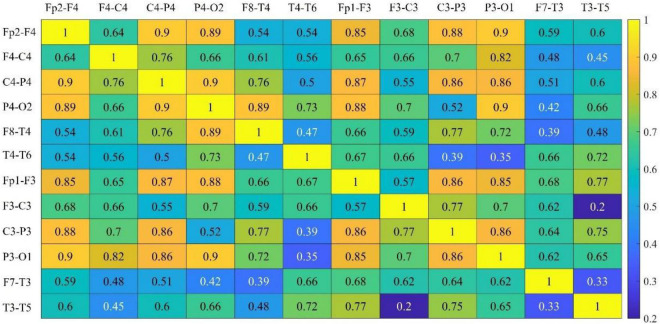
Communication strength of all channels.

The channel selection proposed in Section “3.2. Multi-subsegment strategy” has been tested. In Figure 4, the connectivity coefficients of double channels and all combinations have been separately calculated. In Table 2, the best performance has been obtained when six channels are selected.

**TABLE 2:** Selection of different channels and running time of every 100 events.

Channel selection	Acc (%)	Time/100 events
C4-P4, P4-O2, C3-P3, P3-O1	79.8	1.62 s
Fp2-F4, C4-P4, P4-O2, Fp1-F3, C3-P3, P3-O1	83.2	2.27 s
Fp2-F4, F4-C4, C4-P4, P4-O2, F8-T4, Fp1-F3, C3-P3, P3-O1	81.7	3.11 s
Fp2-F4, F4-C4, C4-P4, P4-O2, F8-T4, Fp1-F3, F3-C3, C3-P3, P3-O1, F7-T3	77.6	4.31 s
All channel	73.6	5.25 s

To reduce algorithm complexity, irrelevant channels or noisy channels have been eliminated, the feature dimension has been reduced. The calculation burden have been reduced. The algorithm operation efficiency and algorithm performance have been improved.

To evaluate the effectiveness of the method proposed in this manuscript, feature extraction is performed directly on the 30 s EEG data using the feature extraction method described in Section “2. Materials.” The results obtained by inputting the features into the classifier can demonstrate the classification performance of the PLV as a feature, and the classification results can be further compared with the classification results of the proposed multi-sub feature learning. In addition, the evaluation metrics used in this manuscript include accuracy (Acc), sensitivity (Recall), positive predictive value (Ppv), and F1 score (F1). Based on the experimental data in the second part, the five frequency ranges of δ (0.5–4 Hz), θ (4–8 Hz), α (8–13 Hz), β (13–30 Hz), and γ (30–40 Hz) are tested, respectively. A 5-category sleep staging task is tested. The specific classification results of these five frequency bands are shown in Table 3.

**TABLE 3:** The classification results of five types of sleep stages by directly extracting features from 5-band EEG.

		N1	N2	N3	W	*R*
δ (0.5–4 Hz)	Recall	0.8	0.67	0.5	0.52	0.47
	Ppv	0.72	0.56	0.51	0.66	0.5
	F1	0.76	0.61	0.5	0.58	0.48
	Acc (%)	59.4%				
θ (4–8 Hz)	Recall	0.95	0.75	0.53	0.63	0.73
	Ppv	0.9	0.66	0.73	0.76	0.59
	F1	0.93	0.70	0.62	0.69	0.65
	Acc (%)	72%				
α (8–13 Hz)	Recall	0.71	0.82	0.78	0.75	0.83
	Ppv	0.79	0.82	0.76	0.75	0.67
	F1	0.66	0.82	0.77	0.75	0.74
	Acc (%)	77.8%				
β (13–30 Hz)	Recall	0.55	0.87	0.65	0.63	0.8
	Ppv	0.61	0.70	0.78	0.79	0.65
	F1	0.58	0.78	0.71	0.70	0.72
	Acc (%)	70.3%				
γ (30–40 Hz)	Recall	0.6	0.83	0.52	0.73	0.77
	Ppv	0.9	0.71	0.61	0.76	0.57
	F1	0.72	0.77	0.56	0.75	0.65
	Acc (%)	69.4%				

**FIGURE 5:**
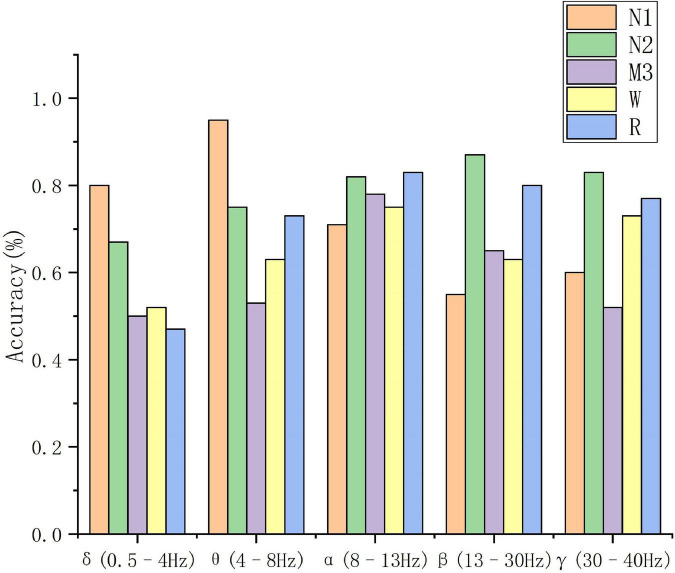
The results of the 5-band EEG direct feature extraction for 5 types of sleep stages.

According to the sleep staging results in Table 3, the overall classification accuracy of the five frequency bands can be obtained as 59.4, 72, 77.8, 70.3, and 69.4%, respectively. It can be seen that the accuracy of the α (8–13 Hz) frequency band (Acc), sensitivity (Recall), positive predictive value (Ppv), F1 score (F1), and other evaluation indicators are better than other frequency bands. On the test set, the α (8–13 Hz) frequency band has the highest classification accuracy for EEG signals, which is 75%, and the δ (0.5–4 Hz) frequency band has the lowest classification accuracy for EEG signals, which is 59.4%. The specific situation is shown in Figure 5.

The classification performance of these five frequency bands on classification tasks is comprehensively analyzed. The classification results of N1 in the θ (4–8 Hz) frequency bands are significantly higher than other frequency bands. The classification accuracy is better than other frequency bands.

### 4.2. Sub-period feature fusion

Based on the abovefive classification tasks for PLV to realize sleep signal, the features obtained by PLV in multiple sub-periods are subjected to feature fusion and are compared with the features extracted from the original signal. The raw EEG is divided into multiple consecutive signal sub-periods that do not overlap, while each piece of raw data uses a different wavelet number to obtain different classification performances. The number of sub-periods is 5, 10, 15, 20, and 30 for five experiments, the number of segments is not suitable for more than 30, because the duration of the K-complex wave and spindle wave needs to be greater than 0.5 s.

The same classification model is used to divide the sleep stages into five classifications, the optimal feature set can be selected for the classification performance. According to the comparison results in Section “3.1. Data preprocessing and channel selection,” the proposed algorithm uses the sleep EEG signal in the α (8–13 Hz) frequency band for experimental testing. The specific experimental results are shown in Table 4.

**TABLE 4:** Accuracy of classification of sleep stages by five frequency bands and five sub-periods under the sub-period feature fusion method.

	δ (0.5–4 Hz)	θ (4–8 Hz)	α (8–13 Hz)	β (13–30 Hz)	γ (30–40 Hz)
5-Sub	63.33%	69.04%	71.73%	70.59%	67.58%
10-Sub	71.67%	75.70%	78.77%	78.20%	72.97%
15-Sub	75.00%	80.59%	79.69%	76.89%	75.68%
20-Sub	78.33%	84.89%	87.00%	81.93%	83.10%
30-Sub	81.67%	86.81%	88.63%	83.82%	81.76%

As shown in Table 4, the accuracy of the multi-segment feature fusion method is 10.83%. The multi-segment feature fusion method can obtain higher classification performance than the method of directly connecting the original signal through brain function. The accuracy of the 30-segment method is the highest, and the accuracy of sleep staging in the α-band reaches 88.63%.

### 4.3. Sub-period decision-making sleep stage classification

**FIGURE 6:**
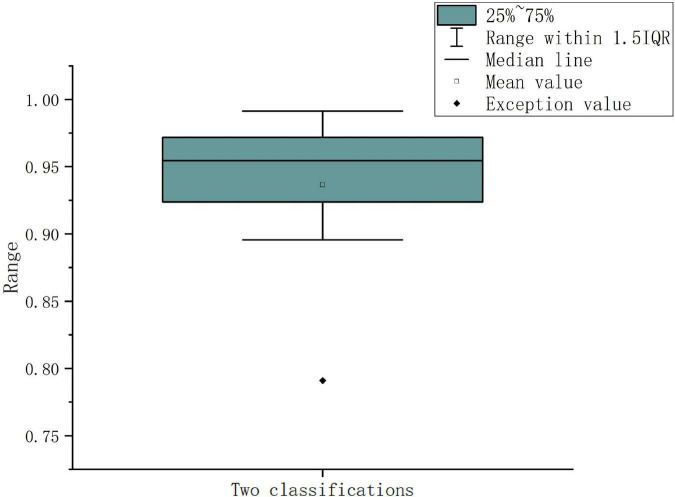
Pairwise classification results for sub-period data.

The sub-period features are extracted according to the optimal frequency band and the number of sub-periods is found in Section “3.2. Multi-subsegment strategy,” the features extracted from sub-periods are directly classified, because the result of the classification is the result of the sub-period, and it cannot represent the category of the original EEG. The divided data are recombined in chronological order. In the combined classification results, the classification result with the most categories is found. As shown in Table 5, it still shows the best classification performance in the α band. The accuracy of 30 sub-period reaches 96.42%. During the experiment, there are cases where the probability of belonging to two or more sleep stages is the same. To solve this problem, the extracted features are input into the binary classifier and the performance of the binary classification is the best. Aiming at the problem of the same probability of sleep stages, the extracted features are re-input to the proposed algorithm to optimize the classification performance strategy and find the final classification result. Figure 6 shows the combination of 5 types of sleep stages, a total of 10 kinds of results, in which the abnormal value appears in the case of N1 Vs W, the classification accuracy is only 79.1%, the average classification accuracy of the remaining 9 combinations is 95.27%, multiple sub-classifiers has chosen to handle this situation. Table 5 shows the classification results of the functionally linked features for different sub-period numbers.

**TABLE 5:** Accuracy of classification of sleep stages in five frequency bands and five sub-periods under sub-period decision-making sleep stage classification method.

	δ (0.5–4 Hz)	θ (4–8 Hz)	α (8–13 Hz)	β (13–30 Hz)	γ (30–40 Hz)
5-Sub	78.33%	84.59%	91.06%	92.02%	81.08%
10-Sub	83.33%	87.82%	91.42%	91.39%	89.19%
15-Sub	83.33%	92.00%	94.27%	92.65%	87.84%
20-Sub	85.00%	92.30%	94.84%	93.28%	90.54%
30-Sub	88.33%	93.04%	96.42%	94.12%	89.19%

### 4.4. Contrast experiment

**FIGURE 7:**
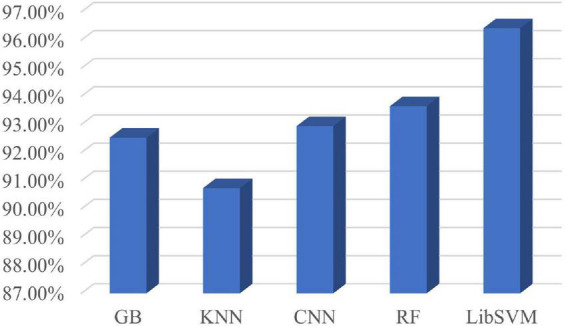
Compare different classifiers.

**FIGURE 8:**
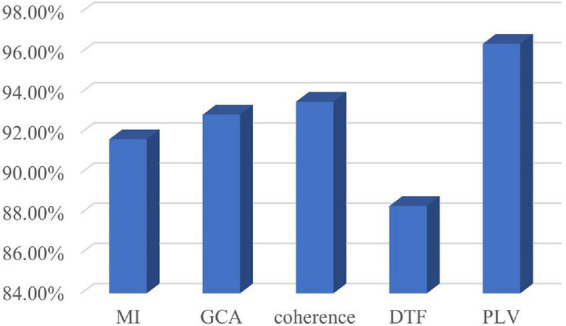
Compare different connection methods.

According to the optimal frequency band and the optimal number of sub-periods found in Section “4.3. Sub-period decision-making sleep stage classification,” a number of comparative tests have been conducted. The comparison includes classification by using various classifiers. The classifiers used in the classification are LibSVM, GB, random forest, k-nearest neighbor (KNN), and CNN. The specific classification results are shown in Figure 7. It can be seen from Figure 7 that the classification effect of LibSVM is better than the other three classifiers as a whole. The accuracy of the method using CNN is low, probably because the extracted features are the features between channels, and the convolution kernel destroys the relative position relationship. Common brain network connection methods include PLV, DTF, coherence, GCA, and MI. The optimal frequency band and the optimal number of sub-periods are analyzed under different connection modes. The concrete results are shown in Figure 8, PLV connection method is better than other methods.

### 4.5. Cross-validation

According to the above experiments, direct PLV feature extraction, multi-sub-period feature fusion, and multi-sub-period decision-making methods are carried out, respectively, and finally, 30 sub-period features are found to have the best classification effect for decision-making. The EEG signals of different subjects have individual differences, the generalization of the proposed algorithm cannot be guaranteed. Therefore, the method needs to be further evaluated for different subjects to test the performance of the method. To demonstrate the effectiveness of the proposed method, the 10-fold cross-validation experiments are conducted between subjects, selecting one subject as the test data and the other subjects as the training set. Since the training dataset and test dataset do not contain EEG samples of the same subjects, cross-validation between subjects can well reflect the generalization of the proposed in practical applications. The α (8–13 Hz) frequency band is used for testing with 30 sub-periods, the final cross-validation results are shown in Table 6. For the experimental results in Table 6, it is found that the final result for the 5-class sleep stage classification is 92.59%, although this result is lower than the result of the random division, the cross-validated method can overcome the randomness of the data.

**TABLE 6:** Sleep classification results after cross-validation.

	N1	N2	N3	W	*R*
Recall	0.96	0.85	0.89	0.96	0.96
Ppv	0.96	0.92	0.86	0.93	0.96
F1	0.96	0.88	0.87	0.95	0.96
Acc (%)	92.59%				

### 4.6. Brain network analysis of PLV

**FIGURE 9:**
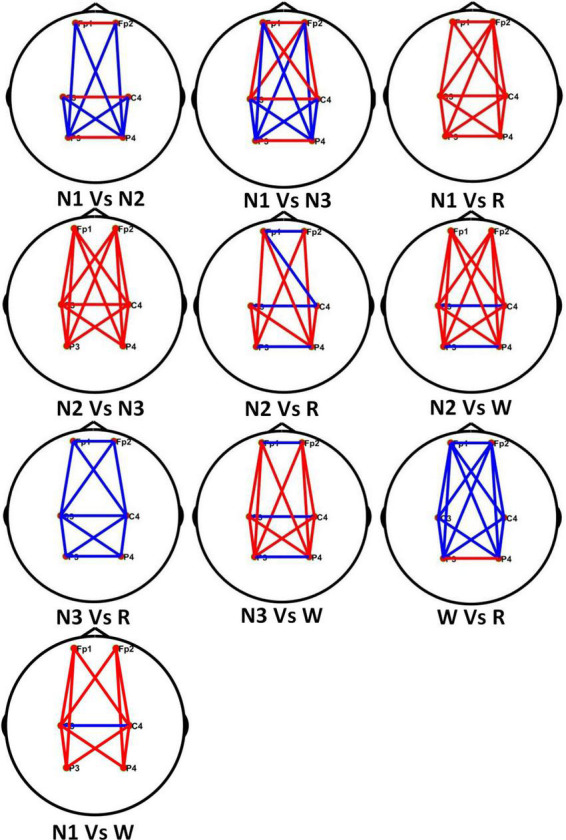
Comparison of brain network connectivity in different sleep stages in theα frequency band.

The PLV values in different stages of the α frequency band of healthy people are combined in pairs. Figure 9 shows the comparison of connectivity in different sleep stages, where the red line represents the former with high connectivity, the blue line is the opposite.

In Figure 9, each line represents the connectivity coefficient between each pair of channels. It can be observed that the overall connectivity of the N1 stage is greater than that of the REM stage, that of the N2 stage is greater than that of the N3 stage, the connectivity of the N3 and W stages is generally smaller than that of the REM stage. The connectivity between the left and right brains in the N1 stage is stronger than that in the N2 stage, but the connectivity between the occipital, parietal, and frontal lobes is weaker than that in the N2 stage. Compared with the N3 stage, the left and right brains of the N1 stage are weaker The connectivity between them is stronger than that in the N3 stage, but the direct connectivity between the frontal and parietal lobes in different brain regions is also greater than that in the N3 stage. For the comparison of N2, W, and REM stages, the connectivity between the left and right brains in the N2 stage is smaller than that in the REM stage, and in the W stage, the connectivity between different brain regions is stronger.

## 5. Discussion

The main purpose of this study is to study the performance of multi-channel EEG signals on sleep staging, obtain effective fusion features using multiple sub-periods, propose a classification optimization strategy, and use the brain function network to analyze the physiological phenomenon of sleep staging. Therefore, a feature learning method of multi-sub-period brain functional network is proposed, which can analyze the features of functional brain network in time series. Comparing the multi-sub-segment and non-multi-sub-segment classification results, the classification accuracy of 30-Sub of 98.63% is significantly better than the original data, and at the same time, it is higher than other classification performances with different numbers of sub-segments. Setting a different number of sub-segments will have different classification effects. For example, in the case of 20-Sub, the classification of the REM stage is better than the division of other sub-segments, which shows that the features extracted in 20-Sub are more suitable for the classification of the REM stage. Different sub-divisions have different classification effects on feature learning, so the relationship between the optimal number of sub-segments and features will be studied in future work. This manuscript explores multi-Sub EEG feature learning for multi-channel EEG sleep staging, which has important potential to improve the application of sleep staging.

Through the analysis of different frequency bands, it is found that the α frequency band has a good performance in classifying sleep stages. For example, in the AASM standard for the classification of sleep stages, the interpretation of the W stage is to record a series of sinusoidal brain waves of 8–13 Hz in the occipital area. The amplitude can be decreased when eyes are open. In addition, α activity may be more pronounced in REM than in N1, the α frequency in REM is usually 1–2 Hz slower than in W. At the same time, the related work of others also have revealed the important role of the α frequency band in sleep staging. Dkhil et al. (2017) proposed the importance of the α band in the assessment of drowsiness. Knaut et al. (2019) found that changes in α oscillations reflect different brain states associated with different levels of wakefulness and thalamic activity. Figure 9 shows the differences in the brain connectivity of different sleep stages in the α frequency band. For example, in stages N2 and N3, the overall connectivity of N2 is greater than that of stage N3. This phenomenon indicates that the connections between brain regions are relatively close. The overall connectivity difference between the N1 and N2 stages is not very obvious, but there is a clear gap in the connectivity between the left and right brain regions.

The proposed framework is compared with the state-of-the-art in sleep stage classification studies, as shown in Table 7. Sharma et al. (2021) recently used the CAP database to decompose EEG epochs into sub-bands using a new class of optimized wavelet filters, the norm features were computed from the six sub-bands coefficients of the optimal wavelet filter bank, which were processed by ensemble of bagged tree (EBT). The ensemble of classifiers obtained 85.3% of unbalanced classification results and 92.8% of balanced data. Tripathy et al. (2020) used 25 subjects for 6 sleep-like stage classification. The CAP database was employed for processing and obtained the classification of 71.68%. This database includes 6 healthy, 7 insomniacs, 1 brux, 1 breathing disorder, and 10 REM behavior disorder patients. Zhao et al. (2022) used CNN to learn the representative features of each sleep stage, feedback on these feature sequences to recurrent neural network (RNN), and learn the context information of sleep stages in chronological order.

**TABLE 7:** Comparison of automatic sleep stage classification algorithms.

References	Description	Performance (%)
Sharma et al., 2021	Database: CAP	Accuracy: (A):92.8 (B):85.3
	Signals: EEG	
	Channel: F4-C4, C4-A1	
	Classification: 6-class A: Balance data B: Imbalance data	
	Features: wavelet decomposition	
	Classifier: EBT	
Tripathy et al., 2020	Database: CAP	Accuracy: 71.68
	Signals: EEG	
	Channel: F4-C4, C4-P4, P4-O2, C4-A1	
	Classification: 6-class	
	Features: Dispersion entropy and Bubble entropy	
	Classifier: Hybrid classifier	
Zhao et al., 2022	Database: CAP	Accuracy: 78.8
	Signals: EEG Channel: C3-A2, C4-A1	
	Classification: 5-class [W vs. S1 vs. S2 vs. (S3 + S4) vs. REM]	
	Features: Convolution feature	
	Classifier: RNN	
Our method	Database: CAP	Accuracy: 92.59
	Signals: EEG	
	Channel: FP2-F4,C4-P4,P4-O2,FP1-F3,C3-P3,P3-O1	
	Classification: 5-class [W vs. S1 vs. S2 vs. (S3 + S4) vs. REM]	
	Features: Interval connectivity coefficient	
	Classifier: LibSVM	

## 6. Conclusion

The framework of dividing sleep stages by multi-sub-segment brain functional connectivity has been proposed. The original EEG signal is filtered into different frequency bands, and the PLV is calculated for the processed data, respectively. The PLV value represents the connectivity coefficient between different channels, the PLV matrix calculated in different sub-periods is used as a feature to find the optimal frequency band according to the performance of sleep stage classification. Then, the filtered data is divided into different numbers of sub-periods, the PLV matrices of the sub-periods are calculated, the features of different numbers of sub-periods are feature-fused, the optimal number of sub-period classifications is found by the classification performance after feature fusion. Finally, the classification performance optimization strategy is used for classification, the brain network is constructed by PLV to explore the mechanism of brain functional connectivity. Firstly, to test the proposed method, extensive experiments have been performed on the sleep dataset CAP. The classification results are tested and analyzed using two test methods, random data partitioning and inter-subject cross-validation. The final results are 96.42 and 92.59%. These results have demonstrated the effectiveness and robustness of the proposed multi-channel EEG sleep staging algorithm. Secondly, the connectivity of N1 stage is larger than that of REM stage, the N2 stage is larger than that of N3 stage, the connectivity of N3 stage and W stage is smaller than that of REM stage. For the comparison of N2, W, and REM stages, the connectivity between the left and right brains in the N2 stage is smaller than that in the REM stage, and in the W stage, the connectivity between different brain regions is stronger. Finally, different numbers of sub-periods have different performances for distinguishing sleep stages. The case of 30-Sub shows good performance, but using 30-Sub in the α band has a higher error rate between N1 and W stages. In the future, multi-channel EEG signals in the CAP database will be planned to classify different sleep disorders, such as insomnia and REM dyskinesia. Furthermore, graph convolutional networks will be employed for automatic sleep stage monitoring to develop an online brain-computer interface system.

## Data availability statement

Publicly available datasets were analyzed in this study. This data can be found here: https://www.physionet.org/static/published-projects/capslpdb/cap-sleep-database-1.0.0.zip.

## Author contributions

FX, JT, SL, and JL had contributed to the conception and design of the study. JZ, ML, and XY had conducted the experiments. CW and YTL had collected the data. WS and YBL have processed the data. LG and BZ had drafted the manuscript. JL had reviewed the manuscript. All authors contributed to the article and approved the submitted version.

## References

[B1] Miyata S. Noda A. Nakata S. Yagi H. Yanagi E. Honda K. (2007). Daytime polysomnography for early diagnosis and treatment of patients with suspected sleep-disordered breathing. *Sleep Breath* 2 109–115. 10.1007/s11325-006-0091-9 17221275

[B2] Younes M. (2017). The case for using digital eeg analysis in clinical sleep medicine. *Sleep Sci.* 1 1–15. 10.1186/s41606-016-0005-0

[B3] Yan R. Zhang C. Spruyt K. Wei L. Wang Z. Tian L. (2019). Multi-modality of polysomnography signals’ fusion for automatic sleep scoring. *Biomed. Signal Process* 11 13–23. 10.1016/j.bspc.2018.10.001

[B4] Ronzhina M. Janoušek O. Koláøová J. Nováková M. Honzík P. Provazník I. (2012). Sleep scoring using artificial neural networks. *Sleep Med. Rev.* 16 251–263. 10.1016/j.smrv.2011.06.003 22030383

[B5] Chapotot F. Becq G. (2010). Automated sleep-wake staging combining robust feature extraction. Artificial neural network classification, and flexible decision rules. *Int. J. Adapt. Control Signal Process* 24 409–423. 10.1002/acs.1147

[B6] Wolpert E. A. (1969). A manual of standardized terminology. techniques and scoring system for sleep stages of human subjects. *Arch. Gen. Psychiatry* 20 246–247. 10.1001/archpsyc.1969.01740140118016

[B7] Berry R. B. Brooks R. Gamaldo C. E. Harding S. Marcus M. C. Vaughn B. V. (2012a). The AASM manual for the scoring of sleep and associated events. Rules, terminology and technical specifications. *Am. Acad. Sleep Med. (Darien)*. Available online at: http://www.aasmnet.org/scoringmanual/

[B8] Diykh M. Li Y. (2016). Complex networks approach for EEG signal sleep stages classification. *Expert. Syst. Appl.* 63 241–248. 10.1016/j.eswa.2016.07.004

[B9] Seo H. Back S. Lee S. Park D. Kim T. Lee K. (2020). Intra-and inter-epoch temporal context network (IITNet) using sub-epoch features for automatic sleep scoring on raw single-channel EEG. *Biomed. Signal Process* 61:102037. 10.1016/j.bspc.2020.102037

[B10] Weber F. D. Supp G. G. Klinzing J. G. Mölle M. Engel A. K. Born J. (2021). Coupling of gamma band activity to sleep spindle oscillations–a combined EEG/MEG study. *NeuroImage* 224:117452. 10.1016/j.neuroimage.2020.117452 33059050

[B11] An P. Yuan Z. Zhao J. (2021). Unsupervised multi-subepoch feature learning and hierarchical classification for EEG-based sleep staging. *Expert Syst. Appl.* 186:115759. 10.1016/j.eswa.2021.115759

[B12] Gunnarsdottir K. M. Gamaldo C. E. Salas R. M. Ewen J. B. Allen R. P. Sarma S. V. (2018). “A novel sleep stage scoring system: combining expert-based rules with a decision tree classifier,” in *Proceedings of the 40th annual international conference of the IEEE engineering in medicine and* biology society (EMBC). Honolulu: 3240–3243. 10.1109/EMBC.2018.8513039 PMC6496951 30441082

[B13] da Silveira T. L. Kozakevicius A. J. Rodrigues C. R. (2016). Single-channel EEG sleep stage classification based on a streamlined set of statistical features in wavelet domain. *Med. Biol. Eng. Comput.* 55 343–352. 10.1007/s11517-016-1519-4 27193344

[B14] Zhu G. Li Y. Wen P. (2014). Analysis and classification of sleep stages based on difference visibility graphs from a single-channel EEG signal. *IEEE J. Biomed. Health Inform.* 18 1813–1821. 10.1109/JBHI.2014.2303991 25375678

[B15] Tabar Y. R. Mikkelsen K. B. Rank M. L. Hemmsen M. C. Kidmose P. (2021). Investigation of low dimensional feature spaces for automatic sleep staging. *Comput. Meth. Programs Biomed.* 205:106091. 10.1016/j.cmpb.2021.106091 33882415

[B16] Mousavi Z. Rezaii T. Y. Sheykhivand S. Farzamnia A. Razavi S. (2019). Deep convolutional neural network for classification of sleep stages from single-channel EEG signals. *J. Neurosci. Methods*. 324:108312. 10.1016/j.jneumeth.2019.108312 31201824

[B17] Khalili E. Asl B. M. (2021). Automatic sleep stage classification using temporal convolutional neural network and new data augmentation technique from raw single-channel EEG. *Comput. Meth. Programs Biomed.* 204:106063. 10.1016/j.cmpb.2021.106063 33823315

[B18] Stevner A. B. A. Vidaurre D. Cabral J. Rapuano K. Nielsen S. F. V. Tagliazucchi E. (2019). Discovery of key whole-brain transitions and dynamics during human wakefulness and non-REM sleep. *Nat. Commun.* 10 1–14. 10.1038/s41467-019-08934-3 30833560 PMC6399232

[B19] Tagliazucchi E. Wegner F. Morzelewski A. Brodbeck V. Jahnke K. Laufs H. (2013). Breakdown of long-range temporal dependence in default mode and attention networks during deep sleep. *Proc. Natl. Acad. Sci. U.S.A.* 110 15419–15424. 10.1073/pnas.1312848110 24003146 PMC3780893

[B20] Tagliazucchi E. Laufs H. (2014). Decoding wakefulness levels from typical fMRI resting-state data reveals reliable drifts between wakefulness and sleep. *Neuron* 82 695–708. 10.1016/j.neuron.2014.03.020 24811386

[B21] Fu M. Wang Y. Chen Z. Li J. Xu F. Liu X. (2021). Deep learning in automatic sleep staging with a single channel electroencephalography. *Front. Physiol.* 12:628502. 10.3389/fphys.2021.628502 33746774 PMC7965953

[B22] Lv J. Liu D. Ma J. Wang X. Zhang J. (2015). Graph theoretical analysis of BOLD functional connectivity during human sleep without EEG monitoring. *PLoS One* 10:106063. 10.1371/journal.pone.0137297 26360464 PMC4567068

[B23] Desjardins M. È Carrier J. Lina J. M. Fortin M. Gosselin N. Montplaisir J. (2017). EEG functional connectivity prior to sleepwalking: evidence of interplay between sleep and wakefulness. *Sleep* 40 4. 10.1093/sleep/zsx024 28204773 PMC5806559

[B24] Terzano M. G. Parrino L. Smerieri A. Chervin R. Chokroverty S. Guilleminault C. (2001). Atlas, rules, and recording techniques for the scoring of cyclic alternating pattern (CAP) in human sleep. *Sleep Med*. 3 187–199. 10.1016/S1389-9457(02)00003-5 14592244

[B25] Goldberger A. L. Amaral L. A. Glass L. Hausdorff J. M. Ivanov P. C. Mark R. G. (2000). PhysioBank. Physiotoolkit, and physionet: components of a new research resource for complex physiologic signals. *Circulation* 101 e215–e220. 10.1161/01.CIR.101.23.e215 10851218

[B26] Berry R. B. Budhiraja R. Gottlieb D. J. Gozal D. Iber C. Kapur V. K. (2012b). Rules for scoring respiratory events in sleep: update of the 2007 AASM manual for the scoring of sleep and associated events. *Sleep Med. J. Clin. Sleep Med.* 8 597–619. 10.5664/jcsm.2172 23066376 PMC3459210

[B27] Brignol A. Al-Ani T. Drouot X. (2012). “EEG-based automatic sleep-wake classification in humans using short and standard epoch lengths,” in *Proceedings of the IEEE 12th International Conference on Bioinformatics & Bioengineering (BIBE).* Larnaca: 276–281. 10.1016/j.cmpb.2012.10.002

[B28] Siettos C. Starke J. (2016). Multiscale modeling of brain dynamics: From single neurons and networks to mathematical tools. *Wiley Interdiscip. Rev. Syst.* 8 438–458. 10.1002/wsbm.1348 27340949

[B29] Rattenborg N. C. Amlaner C. J. Lima S. L. (2020). Behavioral, neurophysiological and evolutionary perspectives on unihemispheric sleep. *Neurosci. Biobehav. Rev.* 24 817–842. 10.1016/S0149-7634(00)00039-7 11118608

[B30] Baptista M. S. Kakmeni F. M. Grebogi C. (2010). Combined effect of chemical and electrical synapses in Hindmarsh-Rose neural networks on synchronization and the rate of information. *Phys. Rev. E.* 82:036203. 10.1103/PhysRevE.82.036203 21230157

[B31] Guo X. Simas T. Lai M. C. Lombardo M. V. Chakrabarti B. Ruigrok A. N. (2019). Enhancement of indirect functional connections with shortest path length in the adult autistic brain. *Hum. Brain Mapp.* 40 5354–5369. 10.1002/hbm.24777 31464062 PMC6864892

[B32] Liu Y. Yan L. Zeng B. Wang W. (2010). “Automatic sleep stage scoring using Hilbert-Huang transform with BP neural network,” in *Proceedings of the 4th International Conference on Bioinformatics and Biomedical Engineering.* Chengdu: 1–4.

[B33] Gao Q. Zhou J. Ye B. Wu X. (2015). Automatic sleep staging method based on energy features and least squares support vector machine classifier. *Int. J. Biomed. Eng. Technol.* 32 531–536. 26485973

[B34] Diykh M. Li Y. Wen P. Li T. (2018). Complex networks approach for depth of anesthesia assessment. *Measurement* 119 178–189. 10.1016/j.measurement.2018.01.024

[B35] Brázdil M. Babiloni C. Roman R. Daniel P. Bares M. Rektor I. (2009). Directional functional coupling of cerebral rhythms between anterior cingulate and dorsolateral prefrontal areas during rare stimuli: A directed transfer function analysis of human depth EEG signal. *Hum. Brain Mapp.* 30 138–146. 10.1002/hbm.20491 17999400 PMC6870726

[B36] Bortel R. Sovka P. (2006). EEG–EMG coherence enhancement. *Signal Process.* 86 1737–1751. 10.1016/j.sigpro.2005.09.011

[B37] Chen B. Ma R. Yu S. Du S. Qin J. (2019). Granger causality analysis based on quantized minimum error entropy criterion. *IEEE Signal Process. Lett.* 26 347–351. 10.1109/LSP.2019.2890973

[B38] Caballero-Gaudes C. Ville D. V. Grouiller F. Thornton R. Lemieux L. Seeck M. (2013). Mapping interictal epileptic discharges using mutual information between concurrent EEG and fMRI. *Neuroimage* 68 248–262. 10.1016/j.neuroimage.2012.12.011 23247187

[B39] Yüce A. B. Yaslan Y. (2016). “A disagreement based co-active learning method for sleep stage classification,” in *Proceedings of the 2016 international conference on systems, signals and image processing (IWSSIP).* Bratislava, ACT: IEEE, 1–4.

[B40] Diykh M. Li Y. Wen P. (2016). EEG sleep stages classification based on time domain features and structural graph similarity. *IEEE Trans. Neural Syst. Rehabil. Eng* 24 1159–1168. 10.1109/TNSRE.2016.2552539 27101613

[B41] Phan H. Andreotti F. Cooray N. Chén O. Y. De Vos M. (2018). “DNN filter bank improves 1-max pooling CNN for single-channel EEG automatic sleep stage classification,” in *Proceedings of the 40th annual international conference of the IEEE engineering in medicine and biology society (EMBC).* Honolulu, ACT: IEEE, 453–456. 10.1109/EMBC.2018.8512286 30440432

[B42] Zhou J. Wang G. Liu J. Wu D. Xu W. Wang Z. (2020). Automatic sleep stage classification with single channel EEG signal based on two-layer stacked ensemble model. *IEEE Access* 8 57283–57297. 10.1109/ACCESS.2020.2982434

[B43] Lachaux J. P. Rodriguez E. Martinerie J. Varela F. J. (1999). Measuring phase synchrony in brain signals. *Hum. Brain Mapp.* 8 194–208. 10.1002/(SICI)1097-019319998:4 10619414 PMC6873296

[B44] Quiroga R. Q. Kraskov A. Kreuz T. Grassberger P. (2010). Performance of different synchronization measures in real data: a case study on electroencephalographic signals. *Phys. Rev. E* 82:036203. 10.1103/PhysRevE.65.041903 12005869

[B45] Chang C. C. Lin C. J. (2000). LIBSVM: A library for support vector machines. *ACM Trans. Intell. Syst. Technol.* 2 1–27. 10.1145/1961189.1961199

[B46] Cortes C. Vapnik V. (1995). Support-vector networks. *Mach. Learn.* 20 273–297.

[B47] Dkhil M. B. Chawech N. Wali A. Alimi A. M. (2017). “Towards an automatic drowsiness detection system by evaluating the α band of EEG signals,” in *Proceedings of the IEEE 15th international symposium on applied machine intelligence and informatics (SAMI).* Herl’any, ACT: IEEE, 000371–000376.

[B48] Knaut P. von Wegner F. Morzelewski A. Laufs H. (2019). EEG-correlated fMRI of human α (de-) synchronization. *Clin. Neurophysiol*. 130 1375–1386. 10.1016/j.clinph.2019.04.715 31220698

[B49] Sharma M. Tiwari J. Acharya U. R. (2021). Automatic sleep-stage scoring in healthy and sleep disorder patients using optimal wavelet filter bank technique with EEG signals. *Int. J. Environ. Res. Public Health* 18:3087. 10.3390/ijerph18063087 33802799 PMC8002569

[B50] Tripathy R. K. Ghosh S. K. Gajbhiye P. Acharya U. R. (2020). Development of automated sleep stage classification system using multivariate projection-based fixed boundary empirical wavelet transform and entropy features extracted from multichannel EEG signals. *Entropy* 22:1141. 10.3390/e22101141 33286910 PMC7597285

[B51] Zhao C. Li J. Guo Y. (2022). SleepContextNet: A temporal context network for automatic sleep staging based single-channel EEG. *Comput. Meth. Prog. Biomed.* 220:106806. 10.1016/j.cmpb.2022.106806 35461126

