# Supplementary figures and images for: Exploration of sleep function connection and classification strategies based on sub-period sleep stages
Source: Front Neurosci. 2023 Jan 25;16:1088116. doi: 10.3389/fnins.2022.1088116 (PMC9906994; doi:10.3389/fnins.2022.1088116)

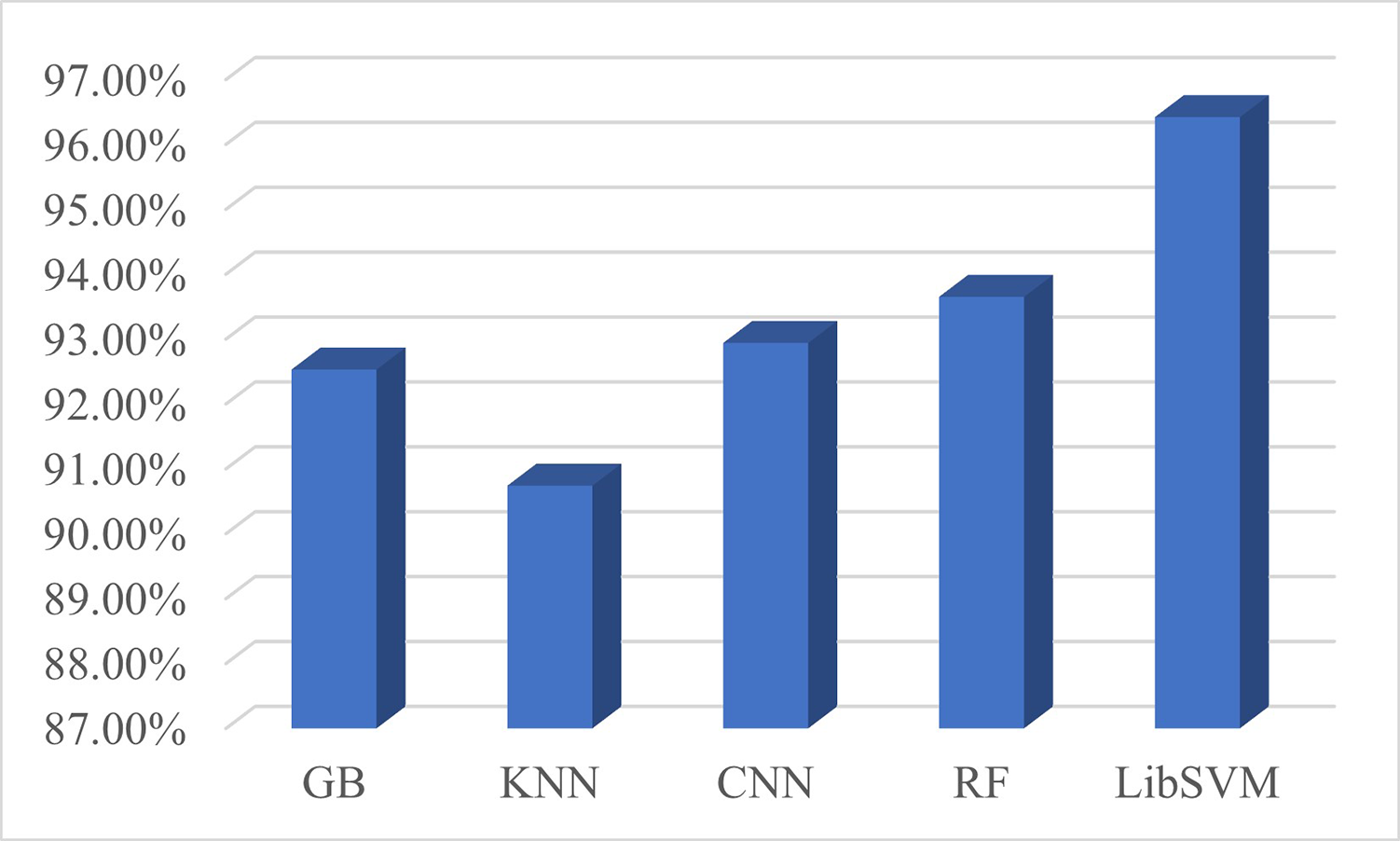

Supplement: Supplementary file 1 [file Image_1.TIF]

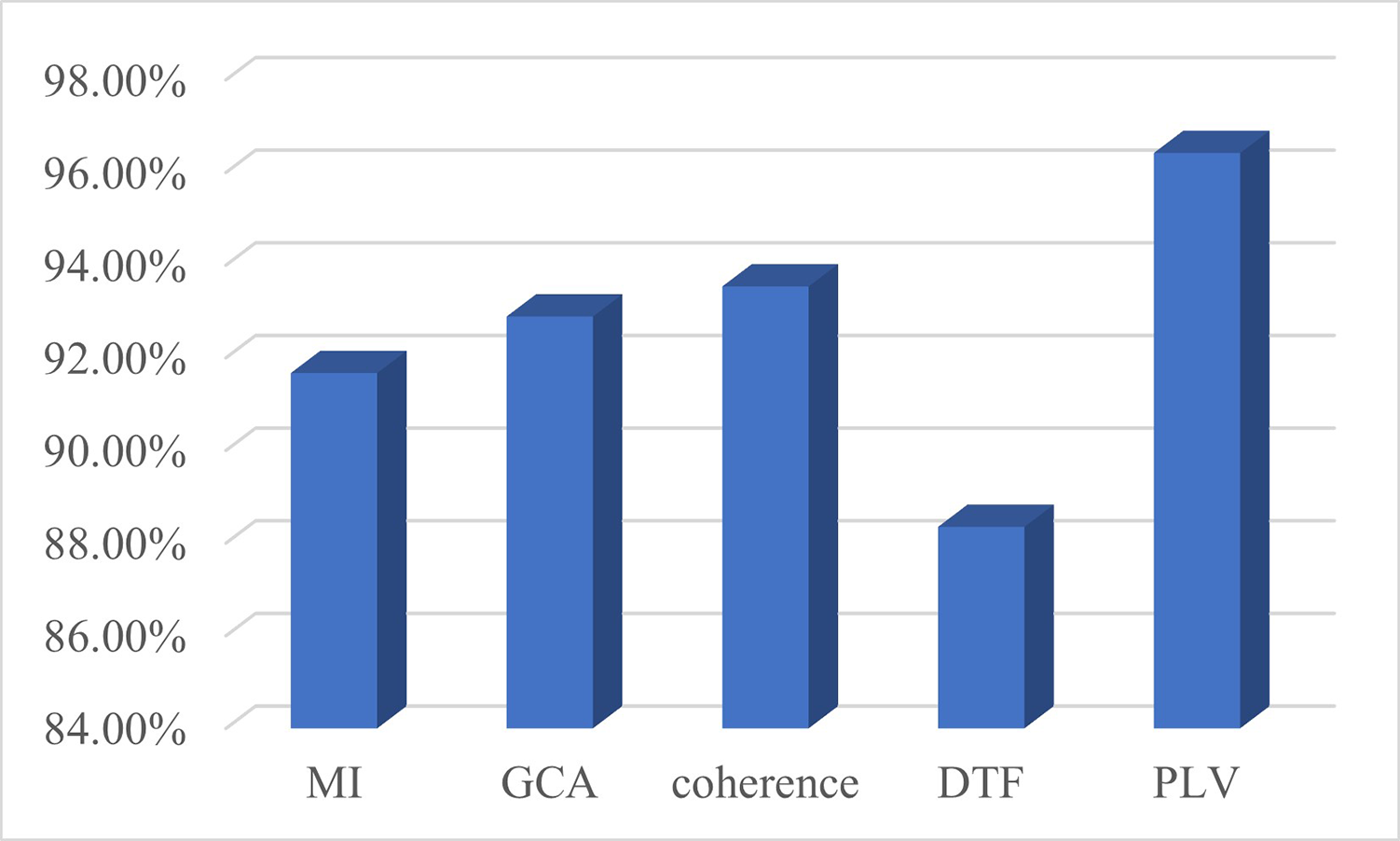

Supplement: Supplementary file 2 [file Image_2.TIF]

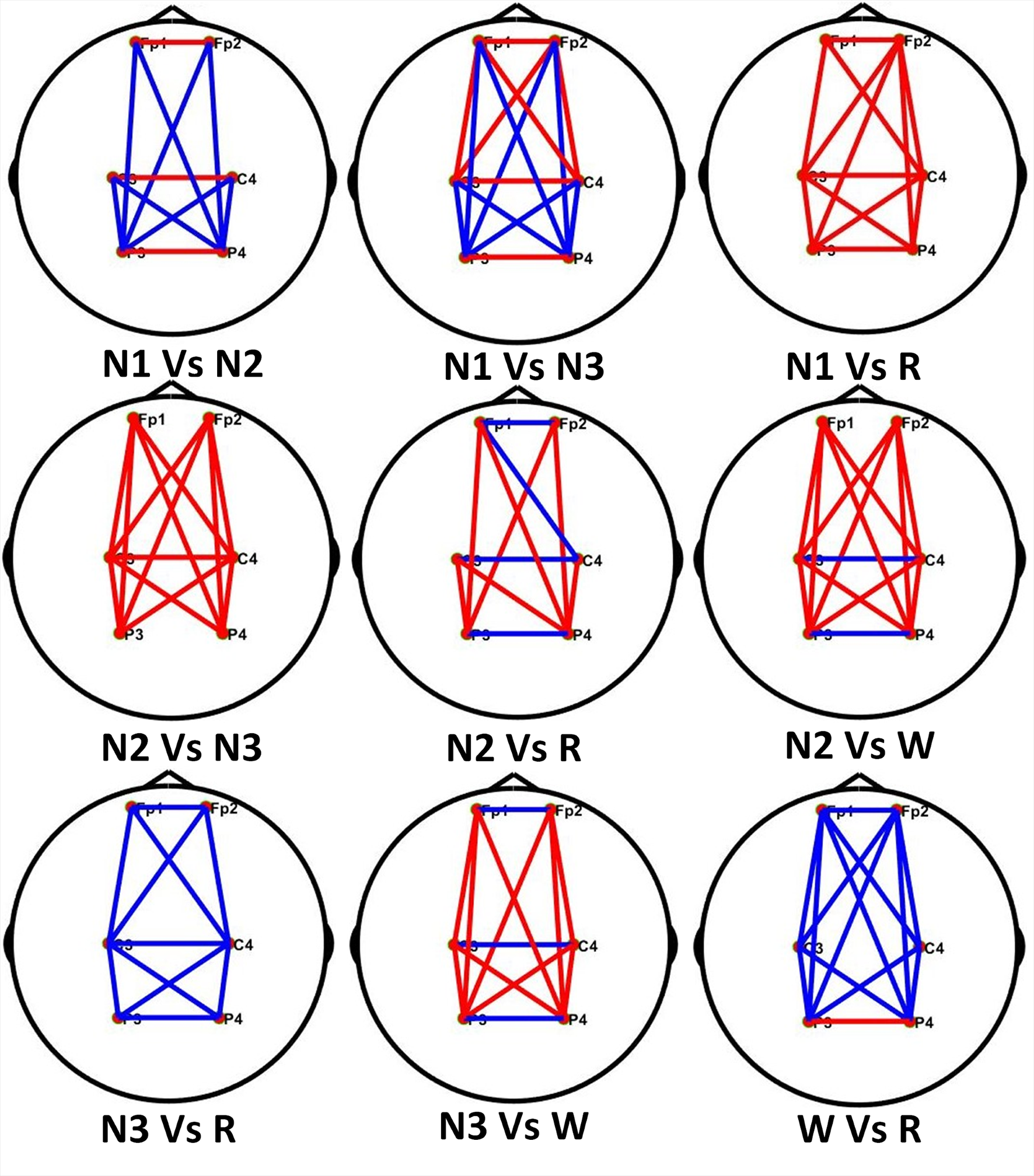

Supplement: Supplementary file 3 [file Image_3.TIF]
